# Genome-wide association study (GWAS) of leaf cuticular wax components in *Camelina sativa* identifies genetic loci related to intracellular wax transport

**DOI:** 10.1186/s12870-019-1776-0

**Published:** 2019-05-07

**Authors:** Zinan Luo, Pernell Tomasi, Noah Fahlgren, Hussein Abdel-Haleem

**Affiliations:** 1 US Arid Land Agricultural Research Center, USDA ARS, Maricopa, AZ 85138 USA; 2 Danforth Plant Science Center, St. Louis, MO 63132 USA

**Keywords:** Leaf cuticular wax, Intracellular wax transport, Genome-wide association studies (GWAS), *Camelina sativa*

## Abstract

**Background:**

It is important to explore renewable alternatives (e.g. biofuels) that can produce energy sources to help reduce reliance on fossil oils, and reduce greenhouse gases and waste solids resulted from fossil oils consumption. *Camelina sativa* is an oilseed crop which has received increasing attention due to its short life cycle, broader adaptation regions, high oil content, high level of omega-3 unsaturated fatty acids, and low-input requirements in agriculture practices. To expand its *Camelina* production areas into arid regions, there is a need to breed for new drought-tolerant cultivars. Leaf cuticular wax is known to facilitate plant development and growth under water-limited conditions. Dissecting the genetic loci underlying leaf cuticular waxes is important to breed for cultivars with improved drought tolerance.

**Results:**

Here we combined phenotypic data and single nucleotide polymorphism (SNP) data from a spring *C. sativa* diversity panel using genotyping-by-sequencing (GBS) technology, to perform a large-scale genome-wide association study (GWAS) on leaf wax compositions. A total of 42 SNP markers were significantly associated with 15 leaf wax traits including major wax components such as total primary alcohols, total alkanes, and total wax esters as well as their constituents. The vast majority of significant SNPs were associated with long-chain carbon monomers (carbon chain length longer than C_28_), indicating the important effects of long-chain carbon monomers on leaf total wax biosynthesis. These SNP markers are located on genes directly or indirectly related to wax biosynthesis such as maintaining endoplasmic reticulum (ER) morphology and enabling normal wax secretion from ER to plasma membrane or Golgi network-mediated transport.

**Conclusions:**

These loci could potentially serve as candidates for the genetic control involved in intracellular wax transport that might directly or indirectly facilitate leaf wax accumulation in *C. sativa* and can be used in future marker-assisted selection (MAS) to breed for the cultivars with high wax content to improve drought tolerance.

## Background

*Camelina sativa* L. Crantz (2n = 40, genome size ~ 782 Mb), belonging to Brassicaceae (Cruciferae) family, is an economically crop originated from southeastern Europe and southwestern Asia [1, 2]. After being cultivated in Europe and North America for centuries until 1950s, *C. sativa* was replaced by another higher-yielding oilseed crop, rapeseed. Recently, *C. sativa* revived public interest due to its exceptional level of omega-3 essential fatty acids, favorable agronomic characteristics, and potential to be a biofuel resource [3]. The oil content in *C. sativa* (36–47%) is twice as that of soybean (18–22%) [4] and its unsaturated fatty acids levels account for over 90% of total oil content, among which omega-3 α-linolenic essential fatty acid can reach up to 40% of total oil content [3]. These oil quality characteristics and advantageous agronomic traits attributes including early maturity [2], low-input requirements for water, nutrients and pesticides [2, 5], broader adaptability to diverse environments [1] and resistance against insects and pathogens [6], make *C. sativa* an ideal alternative resource for biofuel and animal feedstock for the development of sustainable agriculture. Despite these potentials, only limited breeding efforts have been carried out on *C. sativa* and very few registered varieties and advanced breeding lines are available so far due to low genetic diversity discovered within small set of released germplasms [7], which mainly bred for higher yield and/or higher oil composition without interest in abiotic stress resistance. Therefore, in order to breed for *C. sativa,* improvements need to be made in biotic and abiotic tolerance as well as in seed yield, oil content, and fatty acids composition.

Plant cuticle forms the first line in plant defense to protect plants from UV irradiation and water loss [8]. Dehydration avoidance is o of the mechanisms that plant species evolved to reduce plant productivity damage under drought stress, which includes depositing leaf cuticular wax to avoid non-stomatal water loss and improve leaf water retention capacity (LWRC) a common assessment for characterizing drought tolerance in plants [9, 10]. The cuticular wax in plant species consists of several major wax components such as alkanes (ALK), aldehydes (ALD), ketones, primary and secondary alcohols, and wax esters (WE), which are derivatives of very long-chain fatty acids (VLCFAs) [9, 11]. The cuticular wax biosynthesis pathway includes two steps: 1) the fatty acid elongase-mediated extension of the C_16_ and C_18_ fatty acids to VLCFA chains (C_20_-C_34_); 2) the conversion of VLCFAs to the major wax components by the decarbonylation (or the alkane) pathway and the acyl reduction (or primary alcohol) pathway in the endoplasmic reticulum (ER). The alkane pathway mediates the production of ALD, secondary alcohols, ALK, and ketones, while the primary alcohol pathway produces primary alcohols (ALC) and WE. Previous studies showed positive correlations between leaf cuticular wax loads and leaf water retention capacity (LWRC) in plant species such as *Arabidopsis thaliana* [12], maize [8], rice [13], banana [9], creeping bentgrass [14], and mulberry trees [15]. Many genes involved in cuticular wax biosynthesis have been characterized and cloned in *Arabidopsis* [16], maize [8], and rice [13] to reduce water loss under water deficit conditions. In *C. sativa*, the overexpression of an *Arabidopsis* gene (MYB96) conferred drought resistance via cuticular wax accumulation [17]. However, few studies have been conducted to provide a comprehensive characterization of potential genes related to the cuticular wax biosynthesis.

The quantity and compositions of epicuticular wax components vary significantly among different species, genotypes and organs [18]. In *C. sativa*, large content variations in cuticular wax components were found in different genotypes, with ALC, ALK, and WE accounting for 86% of the total wax content, followed by free fatty acids (FA), aldehydes (ALD), alkylguaiacols (AG), and methylalkylresorcinols (MAR), which together accounting for less than 5% of total wax content [19, 20]. Each major wax component has their own predominant constituents. For example, C_24_ (ALC24), C_26_ (ALC26) and C_28_ (ALC28) together accounted for 84% of the total ALC, C_29_ (ALK29), C_32_ (ALK32), and C_35_ (ALK35) were the predominant ALK constituents, accounting for 90% of the total ALK, while C_40_ (WE40), C_42_ (WE42), C_44_ (WE44), and C_46_ (WE46) homologs accounted for the majority in WE [20]. In addition, a moderate to high heritability was also found in major wax components and their constituents [20], demonstrating the possible effectiveness to improve genetic gain of these traits under certain selection pressure.

With the rapid development of next-generation sequencing (NGS) technologies, marker-assisted selection (MAS) can be used to accelerate genetic improvements in breeding programs [21]. QTL mapping identifies putative molecular markers underlying alleles/genes that are controlling quantitative traits, and can be used in MAS. Genome-wide association studies (GWAS) is one of the powerful tools to overcome limitations in traditional QTL mapping and could dissect the genetic architecture of complex traits in crop species [22]. To date, GWAS was used to identify candidate loci associated with various traits in plant species such as *A. thaliana* [23], soybean [24], maize [25], rice [26] and *Brassica napus* [27].

In the current study, we aim to apply GWAS analyses using the algorithm of the Settlement of MLM Under Progressively Exclusive Relationship (SUPER) in a *C. sativa* diversity panel, which consists of accessions originally from different geographical regions, to detect positive alleles (genes) potentially related to major leaf wax components and their constituents. This study could lay a foundation in future molecular breeding efforts to improve drought tolerance in *C. sativa* via improving leaf wax accumulation and reducing water loss under water limitation conditions.

## Results

### Phenotyping in wax biosynthesis related traits

Tomasi et al. [20] completed phenotyping of 50 traits related to cuticular wax components and their constituents in a spring *C. sativa* diversity panel. The total leaf wax content (wax_total) was mainly composed of 8 major wax components: free fatty acids (FA), alkanes (ALK), aldehydes (ALD), alkylguaiacols (AG), methylalkylresorcinols (MAR), wax esters (WE), primary alcohols (ALC), β-sitosterol. These major wax components were composed of 41 predominant constituents (i.e. monomers with different numbers of carbon such as ALC22, ALC24, ALC26, etc.) [20]. As a result, significant positive correlations were found between leaf total wax content (wax-total) and the amount of major wax components with *p*-value smaller than 0.0001 [20]. High heritability was also found in ALC and its predominant constituents (i.e. ALC24, ALC26 and ALC28) as well as for ALK and its predominant constituents (i.e. ALK29, ALK31, and ALK33) [20].

### Population structure and linkage disequilibrium (LD)

**Fig. 1:**
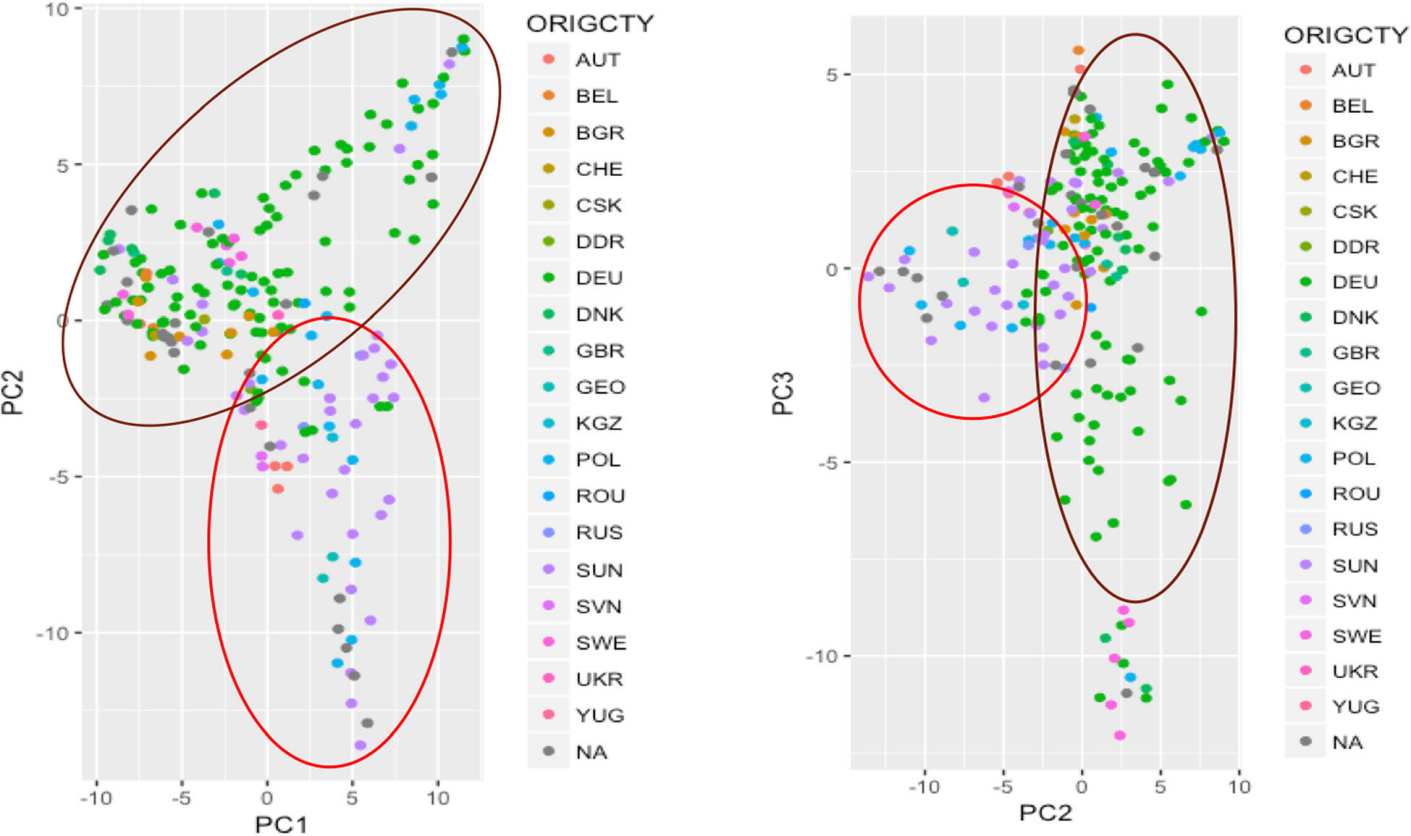
Principle component analysis (PCA) demonstrates two subgroups based on different geographical origins of the spring panel of *Camelina sativa*

The 213 *C. sativa* accessions were sequenced and genotyped using GBS. After sequencing, data processing and SNP filtering, a total of 6192 high-quality SNPs were physically mapped across 20 chromosomes with an average marker density of 101.77 kb per chromosome. Detailed information regarding raw reads, filtered reads, filtered SNPs were provided by Luo et al. [28]. Population structure analysis [28] showed a sharp peak at K = 2 when the number of clusters (K) was plotted against ΔK, meaning that the optimal number of clusters was 2 and the population could be clustered into two subpopulations. In accordance with the population structure analysis, principle component analysis (PCA) results also showed two clearly divergent groups (Fig. 1). Basic statistics results for the two subpopulations regarding major wax components were provided in Table 1 that showed the variation in leaf wax traits between the two geographically separated subpopulations. Linkage disequilibrium (LD) decay varied across chromosomes ranging from 1736 to 3885 kb at r^2^ < 0.2 (Table 2). The average pairwise r^2^ and LD decay at r^2^ < 0.2 for the entire genome was approximately 0.07 and 2591 kb, respectively. The distribution of r^2^ with respect to the physical distance for each chromosome is presented in Table 2. The slowest LD decay was observed for chromosome 10 (3885 kb), followed by chromosome 18 (3599 kb) and chromosome 12 (3364 kb).

**Table 1:** Summary table of basic statistics among two subpopulations and significance of differences in eight major wax components and leaf total wax content

	Pop1						Pop2					
	Min	Max	Median	No.	Mean	SD	Min	Max	Median	No.	Mean	SD
FA_total	0.96	4.65	2.26	55	2.39	0.84	0.91	3.20	2.06	70	2.09	0.63
ALK_total	23.10	119.00	44.40	55	46.90	15.10	25.50	70.50	42.60	70	43.70	9.23
ALC_total	52.50	119.00	75.10	55	75.10	10.80	51.50	93.20	67.30	70	69.50	8.85
ALD_total	0.13	1.44	0.59	55	0.64	0.30	0.13	1.17	0.54	70	0.56	0.25
AG_total	1.96	7.77	3.22	55	3.56	1.21	1.55	7.13	3.81	70	3.93	1.20
MAR_total	1.20	4.30	2.27	55	2.32	0.62	0.98	4.67	2.78	70	2.77	0.75
WE_total	33.40	108.00	53.30	55	56.30	15.50	32.90	80.40	53.20	70	54.00	10.40
Sitosterol	0.06	0.88	0.19	55	0.24	0.17	0.06	0.64	0.20	70	0.24	0.15
Wax_total	152.00	288.00	200.00	55	205.00	29.10	158.00	250.00	194.00	70	195.00	22.60

**Table 2:** Details of LD Decay distance observed at R^2^ < 0.2 on different chromosomes and the entire genome in *Camelina sativa*

Chromosome	Size (Mb)	Mean r2	LD decay distance (Kb) at r2 < 0.2	Count
1	22.86	0.06	2462.534	3506
2	27.50	0.06	3094.724	2885
3	28.19	0.08	2152.146	3974
4	29.72	0.07	2773.029	4081
5	34.76	0.05	2914.815	5176
6	26.00	0.06	2766.698	2922
7	33.05	0.06	2401.861	6601
8	27.63	0.06	2068.315	6793
9	37.52	0.04	2401.333	4918
10	25.04	0.07	3885.220	1672
11	49.58	0.07	3038.294	8268
12	32.05	0.13	3364.167	5533
13	23.49	0.08	1736.596	4044
14	31.47	0.05	2383.700	6093
15	29.96	0.06	2179.102	4503
16	28.91	0.08	2193.280	6621
17	34.94	0.09	2693.883	4034
18	20.62	0.07	3599.897	2294
19	25.84	0.10	1793.181	5132
20	29.83	0.05	1927.620	5473
Whole genome	598.96	0.07	2510.551	102,614
Average	29.95	0.07	2591.520	4726

### Genome-wide association analysis (GWAS) in *Camelina sativa*

**Fig. 2:**
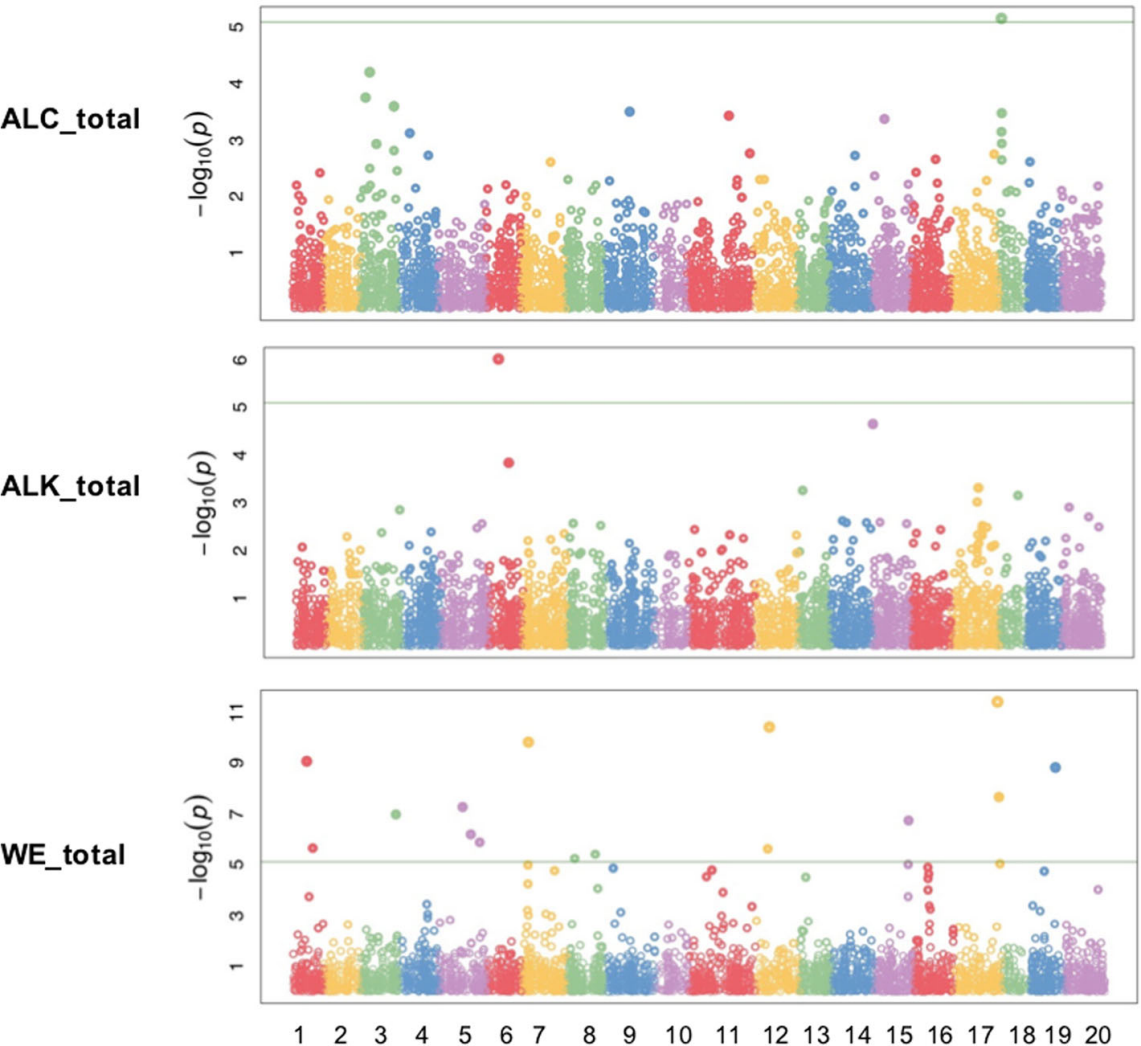
Manhattan plots of GWAS results showing significant SNPs associated with total wax esters (WE_total), total alkanes (ALK_total) and total primary alcohols (ALC_total) in spring *Camelina sativa* diversity panel. *X-axis shows the distribution of SNPs across 20 chromosomes while y-axis shows Bonferroni corrections threshold. The SNPs in triangle, rectangular and circle shared significance among different trait*

A core set of 125 *C. sativa* accessions were selected for GWAS analyses. In order to reduce false positive rates and improve computational power, GAPIT MLM_SUPER was used to perform analysis. Bonferroni correction was used to retain significant SNPs with the *p*-value smaller than 8.0e-6. A total of 42 SNP markers were significantly associated with 15 out of 50 phenotypic traits. Among eight major wax components, three of them (WE_total, ALK_total, and ALC_total) generated significant SNP hits. Sixteen SNPs, one SNP and one SNP were significantly associated with WE_total, ALK_total and ALC_total, respectively (Fig. 2, Table 3). However, no SNP was significantly associated with FA and its constituents, β-sitosterol as well as total wax content.

**Table 3:** Summary genome-wide association study (GWAS) of loci significantly associated with different leaf major wax components and their constituents in a spring *Camelina sativa* diversity panel (Please see the independent excel table)

SNP	Chr.	Position	Alleles	P.value	Bonferroni Corrected	MAF	FDR Adjusted *P*-values	Nearest Gene	Gene Annotation	Protein Function	Associated Traits
S1_10,108,457	1	10,108,457	A/G	5.64E-10	3.49E-06	0.5281690141	0.00000	LOC104786434	clavaminate synthase-like protein At3g21360	metal ion binding; oxidoreductase activity	WE48
				3.17E-10	1.96E-06		0.00000				WE46
				1.12E-06	6.93E-03		0.00346				WE44
				2.74E-11	1.69E-07		0.00000				WE42
				8.99E-10	5.57E-06		0.00000				WE_total
S1_14,592,616	1	14,592,616	G/A	2.24E-06	1.39E-02	0.1150234742	0.00106	LOC104707605	uncharacterized	NA	WE_total
				6.92E-07	4.28E-03		0.00043				AG19
S3_25,592,310	3	25,592,310	T/C	1.07E-07	6.61E-04	0.0915492958	0.00008	LOC104778100	uncharacterized	NA	WE_total
				3.89E-09	2.41E-05		0.00001				AG19
S5_17,084,259	5	17,084,259	A/C	3.46E-06	2.14E-02	0.9319248826	0.00513	LOC104789323	uncharacterized	NA	WE44
				3.49E-09	2.16E-05		0.00001				WE42
				5.48E-08	3.40E-04		0.00005				WE_total
				3.75E-07	2.32E-03		0.00029				AG19
S5_23,163,110	5	23,163,110	A/G	1.08E-06	6.70E-03	0.9084507042	0.00112	LOC104789501	40S ribosomal protein S11–1-like	RNA binding	WE42
				6.48E-07	4.01E-03		0.00036				WE_total
S5_23,163,133	5	23,163,133	A/C	1.08E-06	6.70E-03	0.9084507042	0.00112	LOC104789501	40S ribosomal protein S11–1-like	RNA binding	WE42
				6.48E-07	4.01E-03		0.00036				WE_total
S5_29,786,454	5	29,786,454	T/C	3.25E-09	2.01E-05	0.0704225352	0.00000	LOC104789700	uncharacterized	NA	WE48
				2.31E-08	1.43E-04		0.00003				WE46
				1.32E-06	8.19E-03		0.00068				WE_total
S6_7,999,118	6	7,999,118	A/G	3.82E-08	2.37E-04	0.0046948357	0.00024	LOC104780231	brefeldin A-inhibited guanine nucleotide-exchange protein 5	vesicle trafficking, PAMP-triggered immunity (PTI), effector-triggered immunity (ETI), and salicylic acid (SA)-regulated immunity	ALK33
				9.75E-07	6.04E-03		0.00604				ALK_total
S7_24,153,024	7	24,153,024	C/T	1.20E-09	7.43E-06	0.929577465	0.00000	LOC104704901	probable purine permease 14	NA	AG19
S7_4,441,246	7	4,441,246	T/C	4.65E-06	2.88E-02	0.1384976526	0.00221	LOC109125528	uncharacterized	NA	WE48
S7_4,787,171	7	4,787,171	A/G	7.15E-11	4.43E-07	0.9201877934	0.00000	LOC104700327	B-box zinc finger protein 25-like	negative regulator of seedling photomorphogenesis	WE48
				2.58E-09	1.60E-05		0.00000				WE46
				1.60E-10	9.91E-07		0.00000				WE_total
S7_9,579,071	7	9,579,071	G/T	4.26E-07	2.64E-03	0.117370892	0.00029	LOC104700816	uncharacterized	NA	AG19
S8_12,332,278	8	12,332,278	C/T	5.84E-07	3.62E-03	0.765258216	0.00090	LOC109126081	RNA-directed DNA polymerase homolog	RNA-directed 5′-3′ RNA polymerase activity; RNA-directed DNA polymerase activity	Mar25
S8_21,155,371	8	21,155,371	C/T	4.36E-09	2.70E-05	0.8004694836	0.00000	LOC104709776	glutathione S-transferase T3-like	conjunction of reduced glutathione to a wide number of exogenous and endogenous hydrophobic electrophiles and detoxification against certain herbicides	WE48
				5.08E-08	3.15E-04		0.00004				WE46
				9.12E-07	5.64E-03		0.00112				WE42
				3.98E-06	2.47E-02		0.00164				WE_total
S8_23,054,597	8	23,054,597	T/C	2.07E-06	1.28E-02	0.061032864	0.00107	LOC104709847	cytochrome P450 81D1-like (pseudo)	metal binding	AG19
S8_5,912,798	8	5,912,798	A/C	6.67E-06	4.13E-02	0.9084507042	0.00283	LOC104706385	uro-adherence factor A-like	cell wall anchored protein--erythrocyte binding activity	WE48
				5.83E-06	3.61E-02		0.00226				WE_total
S9_7,943,388	9	7,943,388	A/G	3.68E-06	2.28E-02	0.9014084507	0.00190	LOC104710709	protein arginine methyltransferase NDUFAF7 homolog, mitochondrial	assembly or stability of mitochondrial NADH:ubiquinone oxidoreductase complex	WE48
S11_13,254,172	11	13,254,172	T/G	6.98E-06	4.32E-02	0.0633802817	0.00393	LOC109124846	vacuolar protein sorting-associated protein 25-like	delivery of transmembrane proteins into the lumen of the lysosome for degradation	WE46
S11_47,072,592	11	47,072,592	A/G	1.17E-06	7.27E-03	0.9014084507	0.00073	LOC104726883	peroxisomal membrane protein PEX14	peroxisome movement	WE48
				3.33E-06	2.06E-02		0.01032				ALC32
				3.68E-08	2.28E-04		0.00011				ALC34
				2.61E-07	1.62E-03		0.00081				ALD30
S11_47,072,593	11	47,072,593	A/T	1.17E-06	7.27E-03		0.00073				WE48
S11_49,518,817	11	49,518,817	C/T	4.14E-06	2.57E-02	0.6267605634	0.00513	LOC104727408	GATA transcription factor 5-like	regulation of some light-responsive genes	WE44
S12_10,333,667	12	10,333,667	C/G	3.76E-10	2.33E-06	0.9460093897	0.00000	LOC104731181	glutamate--cysteine ligase, chloroplastic-like	resistance to fungal and bacterial pathogens-regulation of salicylic acid (SA) and phytoalexin (camalexin) production.	WE48
				1.97E-10	1.22E-06		0.00000				WE46
				1.68E-06	1.04E-02		0.00347				WE44
				4.13E-11	2.56E-07		0.00000				WE_total
				1.26E-06	7.80E-03		0.00071				AG19
S12_20,455,684	12	20,455,684	G/A	2.72E-07	1.68E-03	0.1408450704	0.00056	LOC104732333	uncharacterized	NA	Mar25
S12_9,157,483	12	9,157,483	G/T	2.39E-06	1.48E-02	0.8920187793	0.00106	LOC104731044	kinesin-like protein KIN-7I	somatic cell cytokinesis, cell-plate formation and its expansion	WE_total
S13_1,532,622	13	1,532,622	G/A	6.46E-06	4.00E-02	0.7699530516	0.00400	LOC104734437	probable lysine-specific demethylase ELF6	transcriptional gene regulation; repressor of the photoperiodic flowering pathway and of FT.	Mar25
S13_21,456,713	13	21,456,713	A/G	1.70E-06	1.05E-02	0.7769953052	0.00211	LOC104738457	uncharacterized	NA	Mar25
S13_5,037,723	13	5,037,723	C/A	6.43E-07	3.98E-03	0.0751173709	0.00112	LOC104735215	U-box domain-containing protein 52-like	protein ubiquitination	WE42
S15_11,699,414	15	11,699,414	C/T	2.51E-09	1.55E-05	0.896713615	0.00002	LOC104746477	uncharacterized	NA	Mar25
S15_25,879,428	15	25,879,428	C/T	2.44E-06	1.51E-02	0.9107981221	0.00137	LOC104748799	uncharacterized	NA	WE48
				5.39E-06	3.34E-02		0.00334				WE46
				1.85E-07	1.14E-03		0.00013				WE_total
S16_10,454,057	16	10,454,057	T/C	2.37E-06	1.47E-02	0.1103286385	0.00210	LOC104750305	ERAD-associated E3 ubiquitin-protein ligase HRD1B-like	protein ubiquitination	WE42
S16_11,083,391	16	11,083,391	C/T	8.64E-10	5.35E-06	0.931924883	0.00000	LOC104750339	leucine-rich repeat receptor-like protein CLAVATA2	membrane component	AG19
S16_2,915,369	16	2,915,369	T/C	6.87E-07	4.25E-03	0.1009389671	0.00425	LOC104749495	DEAD-box ATP-dependent RNA helicase 21-like	ribosome biogenesis, mRNA degradation and translation initiation.	ALC26
S16_9,912,650	16	9,912,650	T/C	1.31E-07	8.11E-04	0.112676056	0.00012	LOC104753537	uncharacterized	NA	AG19
S16_9,912,651	16	9,912,651	G/A	7.71E-08	4.77E-04	0.08685446	0.00010	LOC104753537	uncharacterized	NA	AG19
S16_9,912,652	16	9,912,652	G/A	1.31E-07	8.11E-04	0.112676056	0.00012	LOC104753537	uncharacterized	NA	AG19
S17_32,490,691	17	32,490,691	T/A	2.56E-11	1.59E-07	0.0704225352	0.00000	LOC104758647	protein SPA1-RELATED 4-like	Repressor of photomorphogenesis in the blue light	WE48
				7.71E-11	4.77E-07		0.00000				WE46
				1.02E-06	6.32E-03		0.00346				WE44
				4.17E-12	2.58E-08		0.00000				WE_total
				3.65E-07	2.26E-03		0.00226				ALK35
				1.46E-09	9.04E-06		0.00000				AG19
S17_33,558,126	17	33,558,126	A/T	7.83E-10	4.85E-06	0.8849765258	0.00000	LOC104758812	probable acyl-activating enzyme 18, peroxisomal	peroxisomal activation of 2,4-dichlorophenoxybutyric acid (2,4-dB)	WE48
				2.97E-08	1.84E-04		0.00003				WE46
				2.23E-08	1.38E-04		0.00002				WE_total
S17_3,778,452	17	3,778,452	A/G	2.77E-06	1.71E-02	0.882629108	0.00214	LOC104755111	U11NAU12 small nuclear ribonucleoprotein 65 kDa protein	branch-point site recognition for normal plant development	WE42
				5.08E-06	3.15E-02		0.00242				AG19
S18_2,191,451	18	2,191,451	C/A	6.97E-06	4.32E-02	0.3755868545	0.04316	LOC104760305	WUSCHEL-related homeobox 8-like	basal embryo development after fertilization	ALC_total
S19_10,881,936	19	10,881,936	G/C	6.85E-06	4.24E-02	0.103286385	0.00283	LOC104765950	F-box protein At5g07610-like	NA	WE48
				2.56E-06	1.59E-02		0.00176				WE46
				3.63E-06	2.24E-02		0.00249				WE42
S19_19,179,520	19	19,179,520	G/T	4.70E-09	2.91E-05	0.8708920188	0.00000	LOC109131004	histone deacetylase 6-like	protein deacetylase activity; degradation of misfoleded proteins	WE48
				1.61E-07	9.99E-04		0.00012				WE46
				1.57E-09	9.73E-06		0.00000				WE_total
S19_4,005,938	19	4,005,938	C/G	2.19E-06	1.36E-02	0.8544600939	0.00735	LOC104764661	probable DNA helicase MCM8 (pseudo)	DNA repair during meiosis	AG23
				2.21E-06	1.37E-02		0.00228				MAR25
S20_6,023,906	20	6,023,906	G/A	1.59E-08	9.85E-05	0.1408450704	0.00005	LOC104769581	magnesium-chelatase subunit ChlH, chloroplastic	chlorophyll synthesis,plastid-to-nucleus retrograde signaling and abscisic acid (ABA) perception	MAR25

### GWAS for primary alcohols (ALC), aldehydes (ALD) and their predominant constituents

**Fig. 3:**
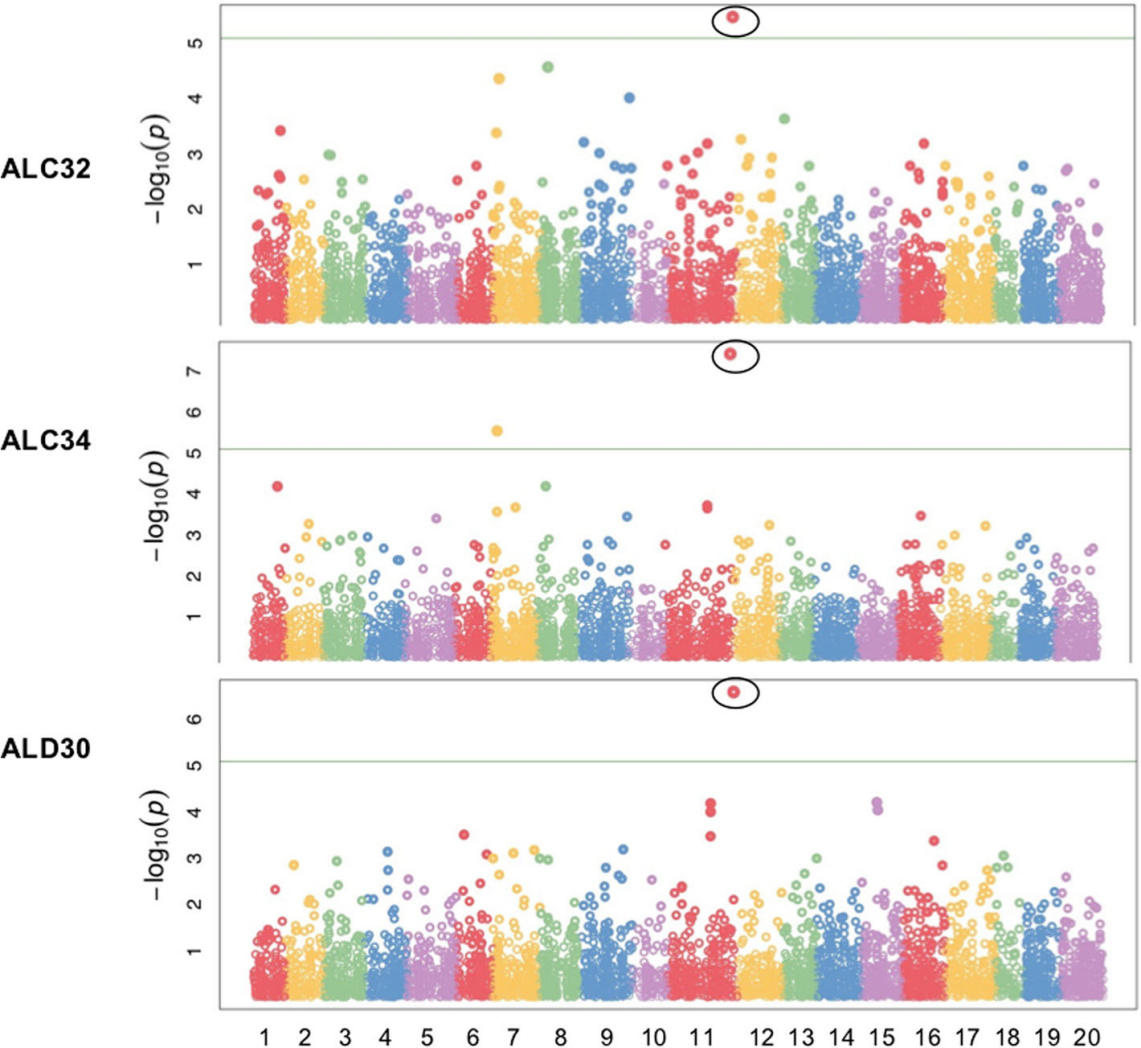
Manhattan plots of GWAS results showing significant SNPs associated with primary alcohols constituents (ALC32 &ALC34) and aldehydes constituents (ALD30) in spring *Camelina sativa* diversity panel. *X-axis shows the distribution of SNPs across 20 chromosomes while y-axis shows Bonferroni corrections threshold. The SNPs in triangle, rectangular and circle shared significance among different traits*

A high heritability (0.77) was found for ALC_total and a moderate to high heritability ranging from 0.38 to 0.86 were found for the predominant ALC constituents as described by Tomasi et al. [20]. ALC_total, ALC26, ALC32 and ALC34 shared the same significant SNP marker on chromosome 11, which was located on a gene functioning peroxisomal import machinery in peroxisome movement, which could contribute to cuticular wax accumulation by maintaining ER network [29] (Fig. 3, Table 3). This SNP was also significantly associated with ALD30 (heritability of 0.53), which was the only constituent under ALD that carrying a significant SNP (Fig. 3, Table 3).

### GWAS for alkanes (ALK) and its predominant constituents

**Fig. 4:**
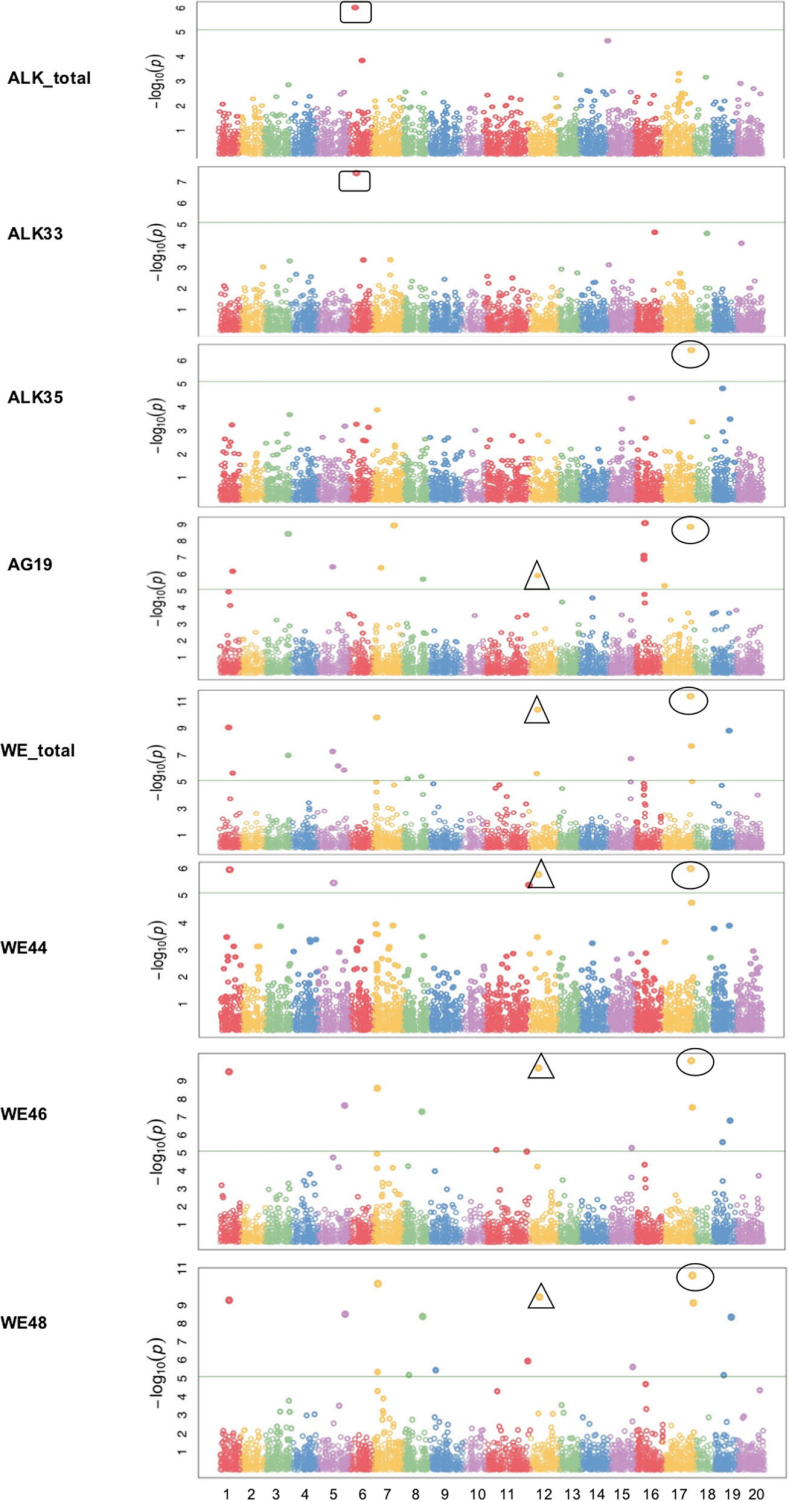
Manhattan plots of GWAS results showing significant SNPs associated with wax esters constituents (WE44, WE46 &WE48), alkanes constituents (ALK33 &ALK35) and alkylguaiacols constituent (AG19) in spring *Camelina sativa* diversity panel. *X-axis shows the distribution of SNPs across 20 chromosomes while y-axis shows Bonferroni corrections threshold. The SNPs in triangle, rectangular and circle shared significance among different traits*

A high heritability (0.77) was observed for total ALK, and a wide range of heritability was estimated for its predominant constituents [20]. ALK_total and ALK33 shared the same significant SNP on chromosome 6 (Fig. 4, Table 3). This SNP was located on ARF-GEF gene, which encodes brefeldin A-inhibited guanine nucleotide-exchange factors (GEF) and activates the auxin response factors (ARF) by exchanging bound GDP for free GTP. This gene plays an important role in vesicle formation and trafficking, which are required for wax secretion from epidermal cells [30–32]. A six-SNP region spanning around the position at 15.68 Mb on chromosome 6 was also found to be associated with ALK_total even if it did not reach the significant threshold level (Fig. 4). Another SNP underlying the gene encoding constitutive photomorphogenic 1 (COP1/SPA) E3 ubiquitin-protein ligase, was significantly associated with ALK35. This SNP was reported to be mainly related to abiotic stress tolerance [33], and its function in photomorphogenesis repressing was also reported [34] (Fig. 4, Table 3).

### GWAS for wax esters (WE) and its predominant constituents

Tomasi et al. [20] estimated a high heritability (0.77) for WE_total and a moderate to high heritability ranging from 0.65 to 0.86 were estimate for six constituents [20]. Sixteen SNPs were identified to be significantly associated with WE_total and some of these SNPs were also associated with WE42, WE44, WE46 and WE48 (Fig. 4, Table 3). These SNPs included three (S17_32,490,691, S11_49,518,817, and S7_4,787,171) that are related to photomorphogenesis repressor, one SNP (S12_10,333,667) that is related to glutamate-cysteine ligase [35], and one SNP (S17_33,558,126) that is related to peroxisomal activation of 2,4-dichlorophenoxybutyric acid (2,4-D), which was reported to increase and maintain water content under citrus fruit epicuticular wax layer during the post-harvest storage period [36]. Some of these SNPs also showed significant associations with other wax sub-constituents, e.g. SNP S17_32,490,691 was found to be significantly associated with both ALK35 and AG19, and SNP S12_10,333,667 was found to be significantly associated with AG19 (Table 3).

### GWAS for alkylguaiacols (AG) and its predominant constituents

A high heritability ranging from 0.77 to 0.87 was found for all the three predominant constituents under AG class [20]. Thirteen SNP and one SNP were significantly associated with AG19 and AG23, respectively. However, most of these SNP neither were located on coding regions (CDS) nor near to characterized genes. Another SNP was found to be significantly associated with AG19, WE_total, and several WE constituents (Fig. 4, Table 3). This SNP is located on glutamate-cysteine ligase, which mainly confers resistance to fungal and bacterial pathogens via the regulation of salicylic acid (SA) and phytoalexin (camalexin) production [37] but also plays an important role in maintaining ER morphology and secretory membrane traffic via glutathione biosynthesis [35]. Other than these, there was a four-SNP region spanning from 9.9 Mb to 11 Mb on chromosome 16 found to be significantly related to AG19 (Fig. 4, Table 3), but no gene functions were annotated in this region.

### GWAS for Methylalkylresorcinols (MAR) and its predominant constituents

**Fig. 5:**
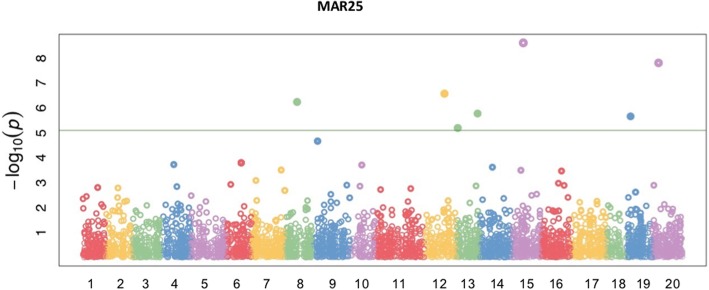
Manhattan plots of GWAS results showing the significant SNPs associated with methylalkylresorcinols constituent (MAR25) in spring *Camelina sativa* diversity panel. *X-axis shows the distribution of SNPs across 20 chromosomes while y-axis shows Bonferroni corrections threshold. The SNPs in triangle, rectangular and circle shared significance among different traits*

Seven significant SNP markers were controlling the phenotypic variations in MAR25 with moderate heritability (0.56). One of the seven SNPs was located on the gene controlling chlorophyll synthesis (Fig. 5, Table 3). Several SNPs, clustered in region spanning 54 bp on chromosome 13 (Fig. 5, Table 3), were positioned on gene encoding lysine-specific demethylase ELF6, which represses the photoperiodic flowering pathway and flowering time [38, 39].

## Discussion

Few molecular breeding and genetics studies have been conducted on *C. sativa* so far. The published genetic/genomic *Camelina* studies were mostly preliminary with small molecular markers coverage that are not enough to be employed in marker-assisted selection. These studies included two genetic maps constructed using recombinant inbred lines (RIL) populations and amplified fragment length polymorphism (AFLP) and simple sequence repeats (SSR) markers [40], and updated with SNP markers [1]. Using these genetic maps, QTLs associated with seed yield, fatty acid compositions and oil content were identified [40]. Recently, a simple marker-trait association study was conducted using 20 winter-type *C. sativa* accessions [41]. On the top of these studies, our study is the first GWAS study in *C. sativa* that based on high-density SNP markers coverage over the 20 chromosomes, and could pave the foundation for marker-assisted breeding in this promising feedstock oilseed crop.

### Population differentiation and linkage disequilibrium (LD)

The number of studies on population genetics of *C. sativa* is limited. Different populations have shown different patterns of population structure and diversity [1, 3, 5, 42], but these studies were based on small population size, small number of molecular markers and/or laborious genotyping technologies. Our results revealed two clearly separated subgroups, which corresponds to the available geographical information in the spring *C. sativa* diversity panel [28]. In general, the extent of LD is affected by the mating system, the breeding history (e.g. the occurrence of bottlenecks) and the genetic diversity in different germplasms and species studied [43]. LD decay is more rapid in outcrossing species and/or germplasms with higher genetic diversity. R^2^ value of 1 in LD means perfect linkage or predictability at one locus with another. The mean pairwise r^2^ value in our study is 0.07, which is greater than *Brassica napus* with a previously estimated mean r^2^ value of 0.037 [44], confirming the higher overall level of LD than *B. napus*. In *C. sativa*, we observed that LD decay below a critical level (r^2^ = 0.2) ranges from 1736 kb to 3885 kb on different chromosomes, with an average value of 2591 kb for the whole genome. These values are greater than *B. napus* where LD decay was between 300 and 1000 kb depending on the germplasm collections [44], or 250 kb in *A. thaliana* [45]. That is not surprising since *C. sativa* is an inbreeding species and most accessions collected were originated from limited regions in Europe and Asia. It was reported that LD decay could vary across different germplasms, for example, LD decay was observed less than 1 kb for maize landraces [46], 2 kb for diverse inbred maize lines [47], and can reach up to100 kb for commercial elite maize inbred lines [48]. Therefore, more *C. sativa* germplasm form different resources are needed to comprehensively compare and estimate LD in *Camelina* species.

### GWAS for major wax components and their constituents

Table 3 shows that the clear majority of traits that associated significantly with SNPs were found to elongate carbon chain longer than C_28_. This was in agreement with a previous finding that significant correlations were found between LWRC and cuticular wax components with carbon chain length longer than C_28_ [9]. These mainly include major components and constituents in ALC (r = 0.730) and WE (r = 0.597) [9]. This may explain why our GWAS study of wax biosynthesis variations resulted in the most majority of significant SNPs associated with ALC and WE components and constituents.

Two ways were hypothesized to form intracellular wax transport: direct transfer from ER to the plasma membrane and Golgi-mediated exocytosis [49]. A few SNPs, located on genes functioning in peroxisomal movement were identified to be significantly associated with wax components and their constituents (Fig. 5, Table 3). This was in agreement with previous findings that peroxisomes help maintain ER morphology and enable its normal functioning in cuticular wax biosynthesis in *Arabidopsis* [29], a closely related species to *C. sativa*.

A SNP located on the gene encoding ARF-GEF was identified to be significantly associated with ALK-related constituents (Fig. 5, Table 3). This is not surprising because ARF-GEFs were reported to act at Golgi membranes, regulating COP1-coated vesicle formation, a function important for ER-Golgi transport [30, 31]. Previous researchers found that very-long-chain (VLC) alkanes, ketones and alcohols synthesized in ER must be trafficked to the plasma membrane to form cuticular waxes and protect plant cuticles [32]. The wax secretion process requires Golgi network-mediated vesicle trafficking, in which ARF-GEFs play an important role. Another SNP (S12_10,333,667) associated with WE_total and its predominant constituents (Fig. 4, Table 3) was related to glutamate-cysteine ligase (GCL), the first enzyme in the glutathione (GSH) biosynthesis pathway. GSH pathway was reported not only to reduce oxidative damage and maintain an intracellular redox environment in response to plant stress [36] but also maintain ER morphology and secretory membrane traffic [35], which were important for intracellular wax transport in cuticular wax biosynthesis [49].

As for a few significant SNPs positioned on genes potentially related to photomorphogenesis repressing or photoperiodic flowering (e.g. COP1/SPA E3 ubiquitin-protein ligase, ELF6, etc.), it remains unknown that whether the light signaling system regulates plant growth and development that has overlapping effects on wax biosynthesis or not. Clustered around significant SNPs that were associated with leaf wax traits, other SNPs, even if not significant, still worthy the investigation with in-depth studies. These SNPs could be located near the genes of functions, or on functioning regulators to mediate the activities of genes. The SNPs with no annotated functions were also likely to be located on novel genes, even if false positive detections may occur. Further validation studies are required to understand the function of these genes or QTL regions.

## Conclusion

This study presents the first GWAS study on *C. sativa*, a promising oilseed crop for food, feed and fuel uses. As many as 50 phenotypic traits related to leaf wax accumulation in Camelina were used to identify putative SNPs associated with these traits. The significant SNPs were positioned in genes directly or indirectly related to cuticular wax accumulation, which might help improve drought tolerance under water deficit. These identified SNPs could provide hints for future molecular breeding studies as potential breakthroughs for the selection of drought tolerance *C. sativa* cultivars. However, more relevant functional genomics, genetics, and validation studies are needed to understand the functions of these alleles/genes.

## Materials and methods

### Plant materials and phenotyping

Phenotypic data of the spring *C. sativa* panel was produced by our research group in 2017 [20]. Briefly, the plant materials we used for current GWAS study came from a spring *C. sativa* diversity panel of *C. sativa* accessions, which consisted of 125 different genotypes and were grown under greenhouse conditions in USDA Arid-Land Agricultural Research Center (ALARC) in Maricopa, AZ in a randomized complete block design (RCBD) with three replications. In brief, the seeds of each accession were planted in pot filled with Sunshine Mix #1/LC1 (Sun Gro Horticulture, Canada), and seedling were regularly watered and fertilized with 20–20-20 fertilizer (Scotts Miracle-Grow, USA). After 35 days of planting, three leaf subsamples were collected from seventh to twelfth leaf of basal rosette. Waxes were extracted following Tomasi el al. [19] protocol with hexane (Sigma-Aldrich, USA), and three internal standards (nonadecanoic acid, tetracosane and tricosano) were added to the hexane-leaves mixture. After 45 s, leaves were removed from the hexane and leaf area were determined using a flatbed scanner and ImageJ software. The hexane extracts including waxes were evaporated to dryness under N2 gas. Samples were re-dissolved in equal amounts of N,O-bis-(trimethylsilyl)-trifluoroacetamide (BSTFA, Sigma-Aldrich, USA) and hexane ad transferred into GC vials. Vials were loaded onto the Agilent 7890A gas chromatograph equipped with a 5975C mass spectrometer Tomasi et al. [19, 20]. Waxes were characterized and quantified by characteristic quadrupole electron impact mass spectra and internal standard and leaf surface areas [19, 20].

### Genotyping-by-sequencing (GBS) of *Camelina sativa* accessions

Briefly as described by Luo et al. [28], DNA extraction was conducted in *C. sativa* lyophilized leaf tissue (~ 0.13 g) using Qiagen Plant DNeasy 96 kit following the manufacturer’s protocol. DNA concentration and quality were determined using Quantifluor (Promega, Inc.) and a Synergy H1 plate reader. The PstI restriction enzyme was used to construct GBS libraries [50]. Library construction and Illumina sequencing were done by the University of Cornell Genomic Diversity Facility. Raw sequence data was analyzed using an HTCondor /Directed Acyclic Graph (DAG) workflow [51] integrated into the TASSEL v5.0 GBS v2 pipeline [52] following the pipeline steps. The HTCondor job files and DAG workflow are available at https://github.com/danforthcenter/camelina. Raw reads were filtered using a minimum base quality score of 20 (kmerLength = 64, minKmerL = 20, mnQS = 20, mxKmerNum = 100,000,000). The quality-filtered reads were aligned to the *C. sativa* genome using BWA MEM [53]. SNPs were called from the alignments with the standards as follows: maxTagsCutSite = 64, mnLCov = 0.1, mnMAF = 0.01. Only biallelic SNPs were filtered by vcftools with missing data smaller than 20% [54]. The VCF file was converted to HAPMAP format using TASSEL. The resulting SNPs were further filtered by disregarding the ones with MAF < 0.05 for GWAS study.

### Population genetic analyses and linkage disequilibrium (LD)

Population structure was estimated using a Bayesian Markov Chain Monte Carlo (MCMC) model implemented in STRUCTURE v2.3.4 [55]. Five independent runs were performed for each assumed population numbers (k) ranging from 1 to 10. Burn-in time and MCMC replication number were both set to 100,000 for each run. Structure Harvester [56] was used to find the most likely K value, which was determined by the log probability of the data (LnP(D)) and ΔK based on the rate of change in (LnP(D) between successive K values [57]. Principle component analysis (PCA) performed in R with the package ggplot2 [58] was also used in population structure analysis. Linkage disequilibrium (LD) between SNPs on each chromosome and on an overall level were estimated using r2 from TASSEL5.0 [52]. For the clustered subpopulations, basic summary statistics were conducted to compare major wax categories: FA_total, ALC_total, ALK_total, ALD_total, AG_total, MAR_total, WE_total, Sitosterol, and Wax_total.

### Genome-wide association studies

Association analyses were performed in R [59] to identify loci controlling wax related traits using the SUPER algorithm [60] implemented in the Genomic Association and Prediction Integrated Tool (GAPIT) package [61]. This method proceeds by extracting a subset of testing SNPs from the total dataset and using pseudo quantitative trait nucleotides (QTNs) that are not in LD with these testing SNPs to define relatedness [60] among the population. This method retains the computational advantage of factored spectrally transformed linear mixed models (FaST-LMM) without impairing the statistical power even when compared to other methods using the entire SNP dataset [60]. To improve the accuracy, we implemented SUPER into mixed linear model (MLM) to perform MLM-SUPER analysis. Manhattan and quantile-quantile (Q-Q) plots were generated using the R package qqman [62]. The Bonferroni correction, negative log (0.05/n), was used as a threshold for significance of associations between SNPs and traits of interests, where n was the total number of SNPs used in the association analysis [57, 63]. Genes within ~ 50 kb upstream and downstream to the associated SNPs were selected for annotation.

### In silico mapping of SNPs and candidate gene identification

Physical mapping of significantly associated SNPs and functional annotation of the predicted genes harboring these SNPs were performed using the *C. sativa* genome browser (https://www.ncbi.nlm.nih.gov/genome/gdv/browser/?context=genome&acc=GCF_000633955.1) submitted by Agriculture & AgriFood Canada. For associated SNPs that mapped to intron regions or gene promoter regions, we included polymorphisms in a 2.0-kb region surrounding the SNP with highest statistical association to the trait. Functional annotation of the genes was performed in the BLAST2GO [64] and UniProt database [65].
